# Mechanisms and management strategies for exacerbated bone loss following denosumab discontinuation

**DOI:** 10.3389/fragi.2026.1886424

**Published:** 2026-07-24

**Authors:** Jiancheng Yang, Ming Yang, Yuhong Zeng

**Affiliations:** Department of Osteoporosis, Honghui Hospital, Xi’an Jiaotong University, Xi’an, China

**Keywords:** bone mineral density, bone turnover, denosumab, discontinuation, fracture, osteoporosis, rebound, sequential therapy

## Abstract

Denosumab is a first-line therapy for osteoporosis, yet the rebound increase in bone turnover markers, rapid bone loss, and elevated fracture risk following its discontinuation have become major challenges in clinical management. This review systematically summarizes the potential mechanisms underlying exacerbated bone loss after denosumab withdrawal and the corresponding sequential treatment strategies. Current mechanistic studies have focused on several hypotheses, including the accumulation of osteomorphs and osteoclast precursors, imbalance in the RANKL/OPG ratio, uncoupling of bone remodeling, and osteocyte-mediated aberrant microdamage repair, which collectively contribute to the burst activation of bone resorption upon treatment cessation. To address this risk, international consensus recommends proactive sequential antiresorptive therapy for patients discontinuing denosumab. For short-term users (≤2.5 years), sequential administration of a single dose of zoledronic acid or alendronate can effectively preserve bone mineral density. For long-term users (>2.5 years), zoledronic acid should be initiated 6 months after the last dose, accompanied by intensive monitoring based on bone turnover markers and repeated dosing as needed. Additionally, emerging strategies such as early transition to romosozumab or alternating use with bisphosphonates have shown potential. Future studies are required to validate the underlying mechanisms in humans and to optimize sequential treatment regimens through prospective trials, thereby enabling individualized therapy and safe discontinuation.

## 1 Introduction

Osteoporosis is a systemic skeletal disorder characterized by reduced bone strength and increased fracture risk, affecting over 200 million patients worldwide. The fragility fractures caused by osteoporosis are among the leading causes of disability and mortality in the elderly, imposing substantial social and economic burdens (Morin et al., 2025). Among pharmacologic agents for osteoporosis, denosumab represents a major milestone in antiresorptive therapy. As the first antibody-based drug approved for osteoporosis treatment, denosumab binds RANKL with high affinity and specificity, mimicking the physiological function of endogenous osteoprotegerin (OPG). This effectively blocks the interaction between RANKL and its receptor RANK, thereby precisely inhibiting osteoclast differentiation, activation, and survival (Lamy et al., 2025). Large-scale randomized controlled trials (e.g., the FREEDOM trial and its extension study) have demonstrated that continuous subcutaneous administration of denosumab 60 mg every 6 months produces nearly linear and sustained BMD gains at the lumbar spine and hip over up to 10 years, with significant reductions in vertebral, non-vertebral, and hip fracture risks (Bone et al., 2017). However, in stark contrast to bisphosphonates—which persist in the bone matrix for extended periods, providing a prolonged “residual effect”—denosumab has a relatively short half-life and does not deposit on bone surfaces. This pharmacological profile dictates that its therapeutic efficacy depends entirely on regular periodic administration. Once treatment is interrupted, the blockade of RANKL is rapidly relieved, and rather than a smooth return to baseline bone turnover, an intense and often overshooting “rebound” of bone resorption ensues (Sølling et al., 2023a). Clinically, this rebound manifests as elevated bone turnover markers, a precipitous decline in BMD, and an increased risk of vertebral fractures (Sendhil et al., 2025). Thus, while denosumab offers remarkable therapeutic efficacy, it simultaneously places its users in a potential “treatment paradox”: having initiated a therapy that may require indefinite continuation, how can it be safely discontinued? A deep understanding of the mechanisms underlying exacerbated bone loss after denosumab discontinuation, coupled with the exploration of effective sequential intervention strategies, has become a frontier research priority and an urgent clinical need in osteoporosis management.

## 2 Risks following denosumab discontinuation

### 2.1 Robust rebound of bone turnover

Bone turnover markers serve as sensitive indicators of early post-discontinuation changes. Upon treatment cessation, the previously suppressed bone resorption and formation activities are rapidly de-repressed. Data indicate that within 3–6 months after the last dose, serum levels of C-terminal telopeptide of type I collagen (CTX), a marker of bone resorption, and procollagen type I N-terminal propeptide (PINP), a marker of bone formation, not only return to baseline but exhibit significant overshoot, with median peak increases of up to 63% for CTX and 47% for PINP above baseline levels (Bone et al., 2011; Miller et al., 2008). This high-turnover state typically persists for 12–24 months before gradually declining toward baseline (Tsourdi et al., 2017). Importantly, the magnitude of bone turnover rebound correlates positively with the duration of denosumab exposure (Everts-Graber et al., 2021). Patients receiving long-term therapy (>3–4 years) may exhibit higher peak CTX levels upon discontinuation, indicating a more pronounced bone resorptive state.

### 2.2 Rapid bone mineral density loss

The decline in BMD following denosumab discontinuation occurs synchronously with the rebound in bone turnover. Studies show that within the first year after discontinuation, the BMD gains achieved at the lumbar spine and total hip during 12 months of treatment are rapidly lost. By the second year, the rate of bone loss slows, and BMD stabilizes at or slightly below pre-treatment levels (Bone et al., 2011; Miller et al., 2008; McClung et al., 2017; Zanchetta et al., 2018). The extent of bone loss after denosumab discontinuation is influenced by multiple factors, including longer denosumab treatment duration, younger age, higher baseline bone turnover, lower BMI, greater BMD gains during treatment, prior fracture history, and no prior antiresorptive therapy (Sølling et al., 2023a; Everts-Graber et al., 2021). A recent study revealed that the relationship between treatment duration and post-discontinuation BMD loss is not simply linear: among patients treated for less than 5 years (10 ± 2 injections), longer treatment was associated with greater bone loss; however, in patients treated for more than 5 years, bone loss reached a plateau (Everts-Graber et al., 2022).

### 2.3 Markedly increased fracture risk

The robust rebound in bone turnover and rapid BMD loss directly translate into an elevated fracture risk, with multiple vertebral fractures being the most prominent feature. Post hoc analyses of the FREEDOM trial and its extension showed that after denosumab discontinuation, the annual incidence of vertebral fractures rebounded from 1.2/100 patient-years during treatment to 7.1/100 patient-years, comparable to the placebo group, but the proportion of multiple vertebral fractures was significantly higher than in the placebo-discontinuation control group (61% vs. 39%, *P* = 0.049) (Cummings et al., 2018). Further analysis revealed that patients who received denosumab for more than 3 years had a higher risk of vertebral fractures after discontinuation (multiple vertebral fractures: 7.5 vs. 2.9/100 patient-years) (Cosman et al., 2022). Real-world evidence corroborates these findings. A study based on a large healthcare database reported hazard ratios of 3.2 for any fracture, 4.7 for vertebral fracture, and 14.6 for multiple vertebral fractures following denosumab discontinuation (Tripto-Shkolnik et al., 2020). An observational study from Switzerland involving 797 patients showed a vertebral fracture incidence of 10.3% (4.1%/year) after discontinuation, significantly higher than the 2.2% (0.75%/year) during treatment (Burckhardt et al., 2021). Prior vertebral fracture, low total hip BMD, elevated bone resorption markers, and greater total hip BMD loss after discontinuation were identified as independent risk factors for fractures.

## 3 Mechanisms of exacerbated bone loss following denosumab discontinuation

**FIGURE 1:**
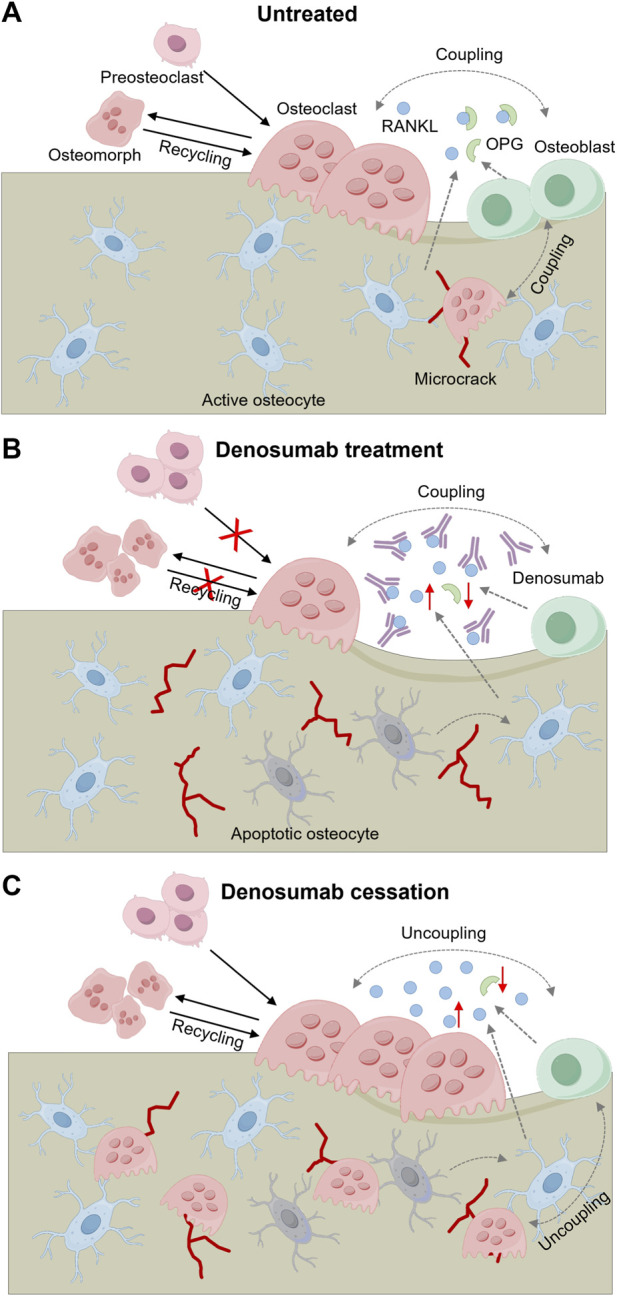
The cascade convergence model underlying exacerbated bone loss following denosumab discontinuation. **(A)** Under physiological conditions, RANKL and OPG are constitutively secreted by osteoblasts and osteocytes, maintaining a low RANKL/OPG ratio. Osteoclasts arise from the differentiation of osteoclast precursors or recycling of osteomorphs. Coupled osteoblasts subsequently refill resorption lacunae with new bone, and microcracks are promptly repaired. **(B)** During denosumab treatment, complete neutralization of RANKL arrests osteoclastogenesis, leading to progressive accumulation of osteoclast precursors and osteomorphs. The coupled reduction in osteoclasts causes a concomitant decline in osteoblasts and decreased OPG output. Suppressed bone resorption permits microdamage accumulation, which triggers peri-lacunar osteocyte apoptosis. Apoptotic osteocytes drive neighboring viable osteocytes to upregulate RANKL secretion; however, this RANKL is neutralized by denosumab, preventing excessive osteoclast activation. **(C)** Upon denosumab discontinuation, the accumulated microdamage and apoptotic osteocytes drive unrestrained RANKL production, while OPG secretion remains suppressed, resulting in a sharp increase in the RANKL/OPG ratio. Accumulated osteoclast precursors rapidly differentiate, and osteomorphs are recycled into numerous active osteoclasts, triggering rapid bone loss. Meanwhile, accumulated microdamage recruits osteoclasts to resorb compromised bone; however, owing to osteoblast-osteoclast uncoupling, osteoblasts fail to promptly deposit new bone, resulting in further net bone loss.

Multiple mechanistic hypotheses have been proposed to explain the exaggerated bone loss following denosumab discontinuation, including RANKL/OPG axis imbalance, accumulation of osteoclast precursors and osteomorphs, osteoblast-osteoclast uncoupling, and osteocyte-mediated microdamage repair impairment. These mechanisms operate at distinct biological levels, from molecular signaling to cellular dynamics to tissue integrity, yet they do not act in isolation. In this section, we present an integrative framework, termed the “cascade convergence model” (Figure 1), in which treatment-induced adaptations at each level converge upon discontinuation to produce a rebound that exceeds pre-treatment baseline. However, it should be noted that the relative quantitative contribution of each proposed mechanism to clinical outcomes, such as vertebral fracture risk and BMD loss, has not been established, and that these mechanisms should be understood as a conceptual framework rather than a quantitatively validated model.

### 3.1 RANKL/OPG imbalance: the molecular engine driving rebound

At the core of the cascade convergence model lies a profound and paradoxical disturbance of the RANKL/OPG axis. Converging evidence from multiple murine models indicates that denosumab and its functional analogs do not merely neutralize existing RANKL but create conditions that amplify RANKL production while diminishing OPG expression, generating a molecular environment primed for explosive osteoclastogenesis upon treatment cessation (Figures 1B,C).

Sølling et al. (2023b) found that serum RANKL levels were significantly increased 6 months after denosumab injection, while OPG levels remained unchanged. Similarly, in mice treated with OPG:Fc (analogous to denosumab), serum RANKL levels increased 17.6-fold compared with controls at the end of 2 weeks of treatment, and further increased 14.7-fold at 6 weeks post-treatment (Kim et al., 2024). Moreover, in OPG:Fc-treated bone, RANKL mRNA expression was significantly higher at 6 weeks after treatment cessation, whereas OPG expression remained comparable to controls. El-Masri et al. (2024) used *in situ* hybridization to identify RANKL-producing cells predominantly on bone surfaces (osteoblasts) and OPG-producing cells mainly in lacunae (osteocytes). They further demonstrated that at the end of 2 weeks of OPG:Fc treatment, Tnfsf11 (encoding RANKL) expression on trabecular bone surfaces was markedly increased, while Tnfrsf11b (encoding OPG) expression in lacunae near cortical bone surfaces was significantly reduced. Fu et al. (2023) generated mice expressing humanized RANKL (huRANKL) and found that 8 weeks of denosumab treatment significantly suppressed OPG expression in osteocytes, subpopulations of OPG-expressing osteocytes located near cortical bone surfaces in both humans and mice. Therefore, after denosumab discontinuation, the accumulated high levels of RANKL are no longer inhibited, while OPG expression remains low, leading to a sharp increase in the RANKL/OPG ratio. This triggers an explosive wave of osteoclastogenesis and activation, resulting in bone resorption far exceeding physiological levels within a short period. It should be emphasized that these mechanistic insights derive predominantly from murine models using OPG:Fc or huRANKL systems, and direct confirmation of equivalent RANKL/OPG dynamics in human bone during denosumab discontinuation remains limited.

The mechanism underlying RANKL accumulation during inhibition remains incompletely understood but likely involves compensatory feedback responses analogous to those observed in endocrine axis suppression. When potent exogenous inhibition blocks RANKL signaling, the skeletal system may upregulate RANKL production in an attempt to restore homeostasis, much as the hypothalamic-pituitary-adrenal axis compensates for exogenous corticosteroids. The duration-dependent nature of the rebound phenomenon finds a plausible mechanistic explanation within this framework. Longer treatment duration likely produces greater cumulative RANKL storage in the bone microenvironment, bound to bone matrix, circulating in serum, and potentially secreted by cells adapting to sustained inhibition, while simultaneously allowing progressive depletion of OPG-expressing osteocytes through treatment-related suppression of their functional activity. Thus, the longer denosumab is administered, the greater the RANKL/OPG imbalance upon withdrawal, potentially explaining the clinical observation that treatment duration positively correlates with rebound severity.

### 3.2 Expansion of the osteoclast lineage pool: two complementary sources

The molecular engine described above would be insufficient to generate the observed rebound without a corresponding expansion of the cellular machinery capable of responding to RANKL signaling. Here, two complementary mechanisms create a dual-source supply system for osteoclasts: the accumulation of osteomorphs, which are transcriptionally distinct daughter cells derived from osteoclast fission, and the expansion of classical osteoclast precursor populations (Figures 1B,C).

The osteomorph concept represents a significant conceptual advance in osteoclast biology. Intravital imaging studies in murine models demonstrated that osteoclasts have an alternative fate to apoptosis following bone resorption: they undergo fission to generate daughter cells expressing 151 genes distinct from both osteoclasts and macrophages, with 40 corresponding knockout mouse lines exhibiting measurable skeletal phenotypes (McDonald et al., 2021). Critically, these osteomorphs retain the capacity to re-differentiate into mature osteoclasts upon RANKL stimulation, whereas RANKL inhibition arrests them in an accumulated state (murine data). The potential clinical relevance of osteomorphs extends beyond murine biology: human homologs of osteomorph-enriched genes show significant association with estimated BMD genome-wide association study (GWAS) loci, suggesting evolutionary conservation of this pathway and potential functional relevance to human skeletal biology. However, the entire osteomorph cascade, from fission to accumulation to re-differentiation, has been documented only in murine models through intravital imaging. Osteomorphs have not been directly visualized or isolated from human bone tissue, their existence in humans remains hypothetical, and no biomarker currently exists to quantify osteomorph-like cells in clinical samples.

The second source of osteoclast supply involves classical osteoclast precursor cells. In a mouse model of anti-RANKL antibody discontinuation, bone marrow analysis revealed marked expansion of osteoclast precursor populations and hematopoietic stem cells following treatment, consistent with a differentiation blockade that permits precursor pool enlargement (Ishikawa et al., 2025). Translating these findings to human biology, studies of postmenopausal women receiving denosumab demonstrated significant increases in circulating CD14+/CD11b+ cells compared with untreated controls (Schini et al., 2024). However, this phenotype requires careful interpretation: CD14+/CD11b+ identifies monocyte populations with multilineage differentiation potential and is not a specific surface marker for osteoclast-committed precursors (Ramchand and McDonald, 2025). The inclusion of additional markers such as CD16 and/or RANK may more precisely define the osteoclastogenic potential of these expanded populations (Xue et al., 2020). Therefore, while the human data support monocyte lineage expansion during denosumab therapy, whether this represents specific osteoclast precursor accumulation remains unresolved.

These two mechanisms likely operate on different temporal scales during the rebound phase. Osteomorphs, being immediately available and transcriptionally primed, may mediate the rapid initial wave of osteoclastogenesis upon treatment cessation, consistent with the observation that tartrate-resistant acid phosphatase (TRAP) activity rises within two to 4 weeks of discontinuation (Kim et al., 2024). Classical precursors, requiring differentiation and maturation, may sustain the prolonged resorptive phase that drives CTX elevation over 3–6 months. This temporal division of labor is entirely hypothetical, based solely on murine observation and indirect biomarker kinetics in humans. Direct evidence distinguishing osteomorph-derived from precursor-derived osteoclast waves in human bone is currently unavailable. Nevertheless, this framework provides a testable model for future investigation.

The dual-source supply model suggests that effective prevention of rebound bone loss may require strategies targeting both immediate (osteomorph-derived) and sustained (precursor-derived) waves of osteoclast generation, potentially informing the timing and mechanism of subsequent antiresorptive therapy.

### 3.3 Osteocyte pathology and microdamage accumulation: the hidden amplifier

The mechanisms described thus far address the supply side of the resorptive equation, RANKL availability and osteoclast lineage expansion. Equally important, however, is the demand signal: what drives the skeletal system to initiate such widespread and aggressive resorption? Emerging evidence points to osteocyte dysfunction and accumulated microdamage as a hidden amplifier that transforms physiological remodeling signals into pathological responses.

Osteocytes fulfill a dual regulatory role in bone homeostasis that becomes profoundly disrupted during prolonged denosumab exposure. First, as the principal source of OPG in the bone microenvironment, osteocytes directly modulate RANKL pathway activity (Fu et al., 2023). Second, through their dendritic network, osteocytes serve as the skeletal mechanosensory system (van Tol et al., 2020), detecting microdamage and initiating targeted repair via regulated apoptosis and local signaling molecule release (Ru and Wang, 2020). Under physiological conditions, microcracks induce focal osteocyte apoptosis; apoptotic osteocytes release RANKL and other osteoclastogenic factors that recruit resorption cells to remove compromised matrix (Cardoso et al., 2009; Verborgt et al., 2009), followed by osteoblast-mediated replacement, an elegant coupled repair sequence that preserves structural integrity.

Prolonged denosumab treatment interrupts this sequence at multiple points. Sustained suppression of bone resorption impedes microdamage repair, permitting progressive accumulation of microcracks throughout the skeleton (Figure 1B). Simultaneously, denosumab-related suppression of osteocyte function reduces OPG production capacity, as demonstrated by the selective loss of OPG expression in peri-surface osteocytes in a huRANKL knock-in mouse model (Fu et al., 2023). Human bone biopsy studies further reveal persistently reduced osteocyte viability and decreased density of viable osteocytes during and after denosumab treatment, suggesting cumulative cellular damage that impairs the bone’s capacity to sense and repair structural compromise (Jähn-Rickert et al., 2020). Notably, the mechanistic link between microdamage accumulation and osteocyte-mediated RANKL release in the context of denosumab discontinuation rests largely on murine data and biomechanical principles; quantitative microdamage assessments in human bone during prolonged antiresorptive therapy remain scarce, and the extent to which accumulated microdamage drives the rebound phenomenon in patients has not been directly established.

When denosumab is withdrawn, the accumulated microdamage, which may be distributed throughout the skeleton due to the systemic nature of treatment, generates a widespread demand signal for bone resorption (Figure 1C). Osteocytes, already compromised in number and function, release large quantities of RANKL in response to microdamage cues (Cardoso et al., 2009; Verborgt et al., 2009). However, because these signals emanate from widespread microdamage rather than focal remodeling needs, the resulting resorptive response is broad and non-selective rather than targeted and coupled. The osteoblast-osteoclast coupling mechanism, which normally ensures that resorption is followed by formation. However, during denosumab treatment, osteoclast suppression concurrently leads to a reduction in osteoblasts. After discontinuation, although osteoclasts rebound rapidly, osteoblast differentiation is delayed, resulting in a temporal mismatch confirmed in murine discontinuation models (Ishikawa et al., 2025). Direct human bone histomorphometric data confirming equivalent uncoupling kinetics during the rebound window are scarce for ethical reasons, and serum biomarkers provide only indirect evidence of this phenomenon in clinical populations.

### 3.4 Osteoblast-osteoclast uncoupling: a temporal mismatch in bone remodeling

A defining feature of the denosumab rebound is not merely the vigor of osteoclast reactivation, but the inability of the bone formation apparatus to mount a matching response. Under physiological conditions, bone resorption and formation are tightly coupled (Figure 1A): osteoclast-derived signals sequentially recruit and activate osteoblast precursors to the resorption site (Bolamperti et al., 2022). This coupling ensures that every quantum of bone removed is eventually replaced. During prolonged denosumab therapy, however, sustained suppression of osteoclast activity reduces the liberation of these matrix-bound osteogenic factors, effectively starving the osteoblast lineage of its primary differentiation cues and diminishing the pool of bone-lining cells available for remodeling activation.

Upon treatment withdrawal, this coupling mechanism fails to re-engage promptly (Figure 1C). In a murine model of anti-RANKL discontinuation, osteoclast numbers on trabecular bone surfaces increased dramatically by 11 weeks post-cessation, while osteoblast numbers remained comparable to control levels; significant osteoblast expansion was not observed until 13 weeks, representing an approximately two-week temporal lag during which bone resorption proceeds unchecked by formation (Ishikawa et al., 2025). This temporal mismatch is mirrored in human biomarker profiles: CTX peaks at approximately 63% above baseline, whereas the concomitant PINP rise reaches only 47% above baseline—a quantitative disparity that translates into net negative bone balance over the critical first 6–12 months after discontinuation (human data) (Bone et al., 2011; Miller et al., 2008).

The uncoupling phenomenon interacts synergistically with the other components of the cascade convergence model. The widespread, non-selective nature of resorption signals generated by accumulated microdamage means that osteoclasts are activated across numerous skeletal sites simultaneously, rather than in the focal, sequential manner characteristic of physiological BMUs. Under normal conditions, each resorption lacuna provides localized osteogenic signals that recruit osteoblasts to the precise site of prior resorption. In the rebound state, the sheer number and dispersal of resorbing surfaces overwhelm this spatial targeting system: osteoblasts, when they do eventually appear, cannot be deployed efficiently to the multitude of sites requiring repair. The result is a transient but clinically consequential state of “remodeling chaos”, intense, diffuse resorption without the structural restoration that normally follows.

However, the uncoupling data derive primarily from murine histomorphometric studies, and direct evidence for equivalent temporal lag in human bone remodeling after denosumab discontinuation is limited. Human bone biopsies during the rebound window are scarce for ethical reasons, and serum biomarkers (CTX, PINP) provide only indirect evidence of uncoupling. Whether the two-week lag observed in mice corresponds to a proportionally longer interval in humans (given the 10–20-fold slower human remodeling rate) remains unknown but clinically relevant.

### 3.5 Translational considerations: what can we conclude from mouse models?

A critical question underlying the entire mechanistic framework presented above is the extent to which murine findings can inform human pathophysiology. The convergence of observations from multiple independent research groups strengthens the internal validity of the proposed mechanisms; however, several fundamental biological differences between mice and humans necessitate careful stratification of conclusions by evidence type and species.

Evidence stratification is essential for interpreting this literature. At the most reliable level, serum biomarker studies in humans confirm that RANKL elevations occur during and after denosumab treatment, and that bone turnover markers overshoot baseline following discontinuation, these observations establish the clinical phenomenon that requires explanation. At an intermediate level, bone histomorphometric studies in mice provide cellular and architectural correlates of the rebound process, including osteoclast-osteoblast uncoupling and microdamage accumulation, these offer plausible but species-dependent mechanistic insights. At the level requiring greatest caution, lineage-tracing studies and single-cell transcriptomic analyses that define osteomorphs and their molecular programs are currently restricted to murine systems, their human relevance remains to be established. The microdamage accumulation hypothesis rests on solid biomechanical principles but requires direct quantification in human bone during prolonged denosumab therapy.

Specific translational limitations deserve explicit enumeration. First, the majority of mechanism studies use young adult mice (typically 6–8 weeks old) with active skeletal growth, whereas denosumab is primarily used in older adults with age-related bone loss, fundamental differences in bone remodeling rates, marrow adiposity, and cellular signaling milieu limit direct extrapolation. Second, murine bone undergoes remodeling approximately 10–20 times faster than human bone, meaning that the temporal dynamics observed in mouse studies compress months of human biology into weeks; the 4-week murine rebound timeline may correspond to 6–12 months in humans. Third, OPG:Fc, the reagent used in several key studies, differs pharmacokinetically from denosumab in both half-life and tissue distribution, and direct comparisons must be made cautiously. Fourth, humanized RANKL knock-in mice, while representing a major technical advance, exhibit altered baseline skeletal phenotypes due to the genetic manipulation itself, potentially confounding treatment response interpretation. Fifth, circulating CD14+/CD11b+ cells in humans are not specific osteoclast precursors, and the claimed expansion of osteoclast-committed progenitors during denosumab therapy remains unproven in human bone marrow.

Future directions to bridge these translational gaps should prioritize: (i) human bone biopsy studies during denosumab treatment and after discontinuation, incorporating single-cell transcriptomics to search for osteomorph-like cell populations; (ii) prospective biomarker studies with serial TRAP, CTX, and PINP measurements to validate the temporal cascade model in clinical populations; (iii) development of more specific circulating osteoclast precursor markers (e.g., CD14+/CD16+/RANK+) for human monitoring; and (iv) studies in aged or ovariectomized murine models that better approximate the target human population.

## 4 Sequential therapy strategies following denosumab discontinuation

Given the bone loss and fracture risk associated with denosumab discontinuation, current international consensus clearly recommends that denosumab should not be stopped lightly unless there are contraindications to continued therapy. For patients who genuinely require discontinuation, sequential antiresorptive therapy should be implemented with close monitoring (Tsourdi et al., 2021). The European Calcified Tissue Society (ECTS) position statement recommends that the choice of sequential regimen should be based on treatment duration: for short-term use (≤2.5 years), alendronate or a single infusion of zoledronic acid may be considered; for long-term use (>2.5 years), zoledronic acid should be administered 6 months after the last denosumab injection, with CTX levels monitored to guide re-treatment (Yen et al., 2025). It should be noted that these recommendations are informed by observational studies, relatively small randomized trials, and heterogeneous protocols across centers; definitive sequential therapy algorithms await confirmation from larger, adequately powered prospective studies.

### 4.1 Sequential therapy after short-term denosumab

After short-term denosumab (≤2.5 years), the bone turnover rebound is relatively modest, the magnitude of bone loss is smaller, and sequential therapy is more effective. Multiple studies have confirmed that a single intravenous infusion of zoledronic acid 5 mg effectively maintains lumbar spine and hip BMD, with some patients requiring no additional treatment for up to 5 years (Anastasilakis et al., 2023). Oral alendronate can similarly preserve the BMD gains achieved with denosumab. The DAPS study showed that after 1 year of denosumab treatment, switching to alendronate for 12 months resulted in no significant decline in lumbar spine or total hip BMD (Kendler et al., 2020). In comparison, raloxifene and risedronate showed poorer efficacy in maintaining BMD after discontinuation, with studies reporting varying degrees of bone loss and even vertebral fractures (Tutaworn et al., 2023; Ha et al., 2022); therefore, these agents are not recommended as first-line sequential therapies. Additionally, sequential teriparatide after denosumab does not prevent the initial BMD decline upon discontinuation and is consequently not recommended as a sequential agent (Leder et al., 2015; Tsai et al., 2017).

### 4.2 Sequential therapy after long-term denosumab

After long-term denosumab (>2.5–3 years), the intensity of the rebound and the magnitude of bone loss increase substantially, and a single dose of bisphosphonate is often insufficient to adequately suppress the bone turnover rebound (Lee et al., 2024; Makras et al., 2021). The ZOLARMAB study demonstrated that even with zoledronic acid treatment, BMD declined by 3%–5% regardless of whether it was administered at 6 months, 9 months, or after bone turnover had already increased, and approximately half of the patients required repeat infusions (Sølling et al., 2020). Laursen et al. (2025) managed 66 patients with an average denosumab treatment duration of 6.7 years according to the ECTS-recommended protocol, using a re-treatment threshold of CTX >0.5 μg/L (the mean reference value for premenopausal women) or BMD decline exceeding the least significant change. Results showed that 80% of patients received at least 2 zoledronic acid infusions within the first year, with BMD reductions significantly smaller than those in historical controls. All mean CTX and PINP levels remained within the premenopausal reference range, and no clinical fractures occurred. This study indicates that a lower CTX re-treatment threshold combined with a repeat-dosing strategy provides superior bone protection for long-term denosumab users. A subsequent study further suggested that among patients receiving an average of 4 years of denosumab, those whose CTX remained above the reference range mean at 6 months after the first zoledronic acid dose, even after a second dose, still experienced a 5% decline in vertebral BMD and a 10% vertebral fracture rate, suggesting that some high-risk patients may require earlier and more intensified intervention (Grassi et al., 2024). It is important to recognize that optimal CTX thresholds for retreatment, the ideal timing of zoledronic acid administration (3 vs. 6 vs. 9 months), and the comparative efficacy of repeated versus single-dose infusion strategies remain active areas of investigation, and the protocols described above should be interpreted as evolving guidance rather than definitive standards.

It is noteworthy that the transition from denosumab to zoledronic acid is associated with a high incidence of acute phase response (APR). In the study by Laursen et al., 30% of patients developed influenza-like symptoms after the first infusion, and 22% experienced similar reactions after the second infusion (Laursen et al., 2025). The ZOLARMAB study reported an APR incidence as high as 61% (Sølling et al., 2020). Studies suggest that the higher bone turnover state after denosumab discontinuation may be associated with APR occurrence. Premedication with acetaminophen or ibuprofen can mitigate but not completely eliminate APR (Wark et al., 2012). Therefore, prophylactic management of APR and patient education are important components of sequential therapy implementation.

### 4.3 Other exploratory strategies

Given that the rebound phase is characterized by concurrent excessive bone resorption and relative insufficiency of bone formation, initiating the dual-acting agent romosozumab at the end of denosumab treatment or immediately after discontinuation, creating a combination of “anti-resorptive + anabolic” effects, may more comprehensively maintain bone metabolic balance. Preliminary studies have shown that transitioning from denosumab to romosozumab preserves the total hip BMD gains achieved during denosumab treatment, further increases lumbar spine BMD, and prevents bone turnover marker levels from rising above baseline (McClung et al., 2021). A recent propensity score-matched cohort study reached similar conclusions (Hong et al., 2025). However, the benefits of sequential romosozumab after denosumab require confirmation through larger-scale prospective randomized controlled trials. At present, this strategy should be regarded as investigational and off-label in most clinical settings; it should not replace guideline-based sequential bisphosphonate therapy outside of controlled research protocols.

Overlapping sequential therapy involves initiating a new therapeutic agent before the previous osteoporosis treatment period has fully concluded. For example, Kumar et al. (2024) treated three patients with severe osteoporosis and multiple fragility fractures by starting romosozumab 3 months after the last dose of denosumab, then resuming denosumab after 6 months of romosozumab. The results showed an increase in lumbar spine BMD of 5%–22%. Similarly, another study administered romosozumab at 6 months versus 3 months after denosumab discontinuation; 90% of patients in the 6-month group had CTX levels exceeding the premenopausal reference mean, compared with only 25% in the 3-month group, suggesting that early administration of romosozumab after denosumab discontinuation may more effectively suppress the bone resorption rebound (Piasentier et al., 2025). Unfortunately, the sample size was too small, and BMD responses were not evaluated. A retrospective study demonstrated that initiating teriparatide within 6 months of the last denosumab injection resulted in more pronounced lumbar spine BMD gains compared with initiation at 6–9 months, and all patients with significant BMD decline were in the 6–9 months initiation group (Kumar et al., 2025). These overlapping and early-transition strategies are supported by very small cohorts, case series, or single-center observations; their generalizability remains uncertain, and they should be considered experimental pending validation in larger controlled studies.

Inspired by these findings, and considering that sequential zoledronic acid at 6 months after the last denosumab dose results in slower bone loss compared with 9 or 18 months (Sølling et al., 2020; Anastasilakis et al., 2021), the question arises whether administering zoledronic acid at 3 months after denosumab discontinuation could better preserve BMD gains, an overlapping sequential approach worth exploring. A recent randomized controlled trial revealed that administering one zoledronic acid infusion after denosumab discontinuation followed by resuming denosumab 1 year later not only prevented bone loss but actually increased lumbar spine BMD (Yen et al., 2025), suggesting that an alternating regimen with zoledronic acid may represent a potential strategy to counter bone loss after denosumab discontinuation. Additionally, Khan et al. (2023) explored a denosumab dose-tapering strategy, finding that reducing the dose from 60 mg to 30 mg every 6 months maintained stable BMD, suggesting that gradual dose reduction may attenuate the magnitude of BMD rebound. However, denosumab is currently available only in a 60 mg pre-filled syringe formulation, making dose tapering practically challenging in clinical implementation. Moreover, dose-tapering data are derived from a single small prospective observational study; this approach remains investigational and is not currently endorsed by any major osteoporosis guideline.

In summary, regardless of the sequential strategy adopted, enhanced monitoring is an indispensable component. For all patients who are planning or have already undergone discontinuation, intensive follow-up during the first year is recommended, with monitoring of bone turnover markers such as CTX and BMD every 3–6 months to dynamically assess the degree of rebound and adjust treatment in a timely manner. Incorporating factors such as treatment duration, BMD response, fracture history, and age into risk assessment models may help identify high-risk individuals for more proactive intervention.

## 5 Summary and outlook

The cascade convergence model presented in this review integrates evidence from murine and human studies into a coherent conceptual framework. However, it should be acknowledged that several component mechanisms, rest primarily on animal model evidence, and their direct applicability to human clinical outcomes requires further validation. The mechanisms underlying denosumab-discontinuation bone loss do not operate in isolation; rather, they constitute a self-propagating, positive-feedback cascade in which each element amplifies the others. Osteomorph and osteoclast precursor accumulation creates a large, immediately recruitable cellular reservoir; the elevated RANKL/OPG ratio provides the decisive activation signal for these cells; uncoupling of osteoblast–osteoclast activity prevents compensatory bone formation; and accumulated microdamage, sensed by compromised osteocytes, drives sustained RANKL release that further fuels resorption. This interplay transforms what might be a transient rebound into a prolonged, exaggerated catabolic state. Nevertheless, substantial uncertainty persists: the relative quantitative contribution of each mechanism in human bone has not been established; the existence of osteomorphs in human tissue remains unconfirmed; direct evidence for osteocyte-mediated microdamage repair impairment in patients is scarce; the threshold duration of denosumab exposure at which these mechanisms become clinically dominant is unknown; and the inter-individual variability in rebound severity cannot yet be explained by any single pathway. In particular, we lack predictive biomarkers that can reliably stratify patients by rebound risk before discontinuation. Closing these gaps will require integrated human studies that simultaneously assess cellular dynamics, matrix-level changes, and biomarker trajectories, moving beyond descriptive pathophysiology toward mechanism-targeted prevention and individualized risk stratification.

Future research should prioritize the following four lines of inquiry, each designed to translate mechanistic insights into measurable clinical benefit. First, serum tartrate-resistant acid phosphatase 5b (TRAP-5b) should be prospectively validated as an early predictive biomarker for rebound risk. Preclinical data indicate that TRAP-5b rises earlier than CTX following RANKL inhibition withdrawal, positioning it as a potential leading indicator of emerging osteoclast activity (Kim et al., 2024). Future prospective cohort studies should longitudinally measure both markers at 3, 6, and 12 months post-discontinuation, applying hierarchical clustering of marker trajectories and correlation with subsequent BMD changes, to determine whether TRAP-5b adds predictive value beyond CTX for guiding the timing of bisphosphonate retreatment. Second, methodologies for identifying osteomorphs in human bone must be developed. Although osteomorphs have been characterized extensively in murine models through intravital imaging (McDonald et al., 2021), their existence in humans remains hypothetical. Single-cell or single-nucleus RNA sequencing of bone marrow aspirates or transiliac biopsies from denosumab-treated patients, combined with spatial transcriptomics to localize osteomorph-signature transcripts within the bone microenvironment, could provide the first direct evidence. If validated, osteomorph abundance could serve as a novel biomarker for individualizing discontinuation protocols. Third, adequately powered randomized controlled trials are needed to compare sequential strategies head-to-head. Key design elements should include: (i) factorial randomization to zoledronic acid at 3 versus 6 months after the last denosumab dose; (ii) a biomarker-driven repeat-dosing arm versus single-dose control; and (iii) a romosozumab-sequencing arm for long-term users. Primary endpoints should encompass not only BMD preservation but also vertebral fracture incidence over at least 24 months, powered to detect clinically meaningful differences in multiple vertebral fracture rates. Fourth, an integrated risk prediction model that combines clinical variables (treatment duration, fracture history, BMD response) with longitudinal biomarker trajectories (CTX, PINP, TRAP-5b) should be developed and externally validated using machine-learning approaches. Such a model would enable clinicians to estimate individual rebound probability at the point of discontinuation, select the optimal sequential regimen, and determine the intensity and frequency of monitoring required, thereby transforming the current empirical approach into a precision-medicine framework for safe denosumab withdrawal.
